# Editorial: Physiological Computing of Social Cognition

**DOI:** 10.3389/fnhum.2019.00326

**Published:** 2019-09-18

**Authors:** Antonio Fernández-Caballero, José Miguel Latorre, Arturo Martínez-Rodrigo, Roberto Rodriguez-Jimenez, Amir Hussain

**Affiliations:** ^1^Departamento de Sistemas Informáticos, Escuela Técnica Superior de Ingenieros Industriales, Universidad de Castilla-La Mancha, Albacete, Spain; ^2^Instituto de Investigación en Informática de Albacete, Universidad de Castilla-La Mancha, Albacete, Spain; ^3^CIBERSAM (Biomedical Research Networking Centre in Mental Health), Madrid, Spain; ^4^Departamento de Psicología, Facultad de Medicina, Universidad de Castilla-La Mancha, Albacete, Spain; ^5^Departamento de Sistemas Informáticos, Escuela Politécnica de Cuenca, Universidad de Castilla-La Mancha, Cuenca, Spain; ^6^Instituto de Tecnologías Audiovisuales de Castilla-La Mancha, Universidad de Castilla-La Mancha, Cuenca, Spain; ^7^Instituto de Investigación 12 de Octubre (i+12), Hospital Universitario 12 de Octubre, Madrid, Spain; ^8^Departamento de Psiquiatría, Universidad Complutense de Madrid, Madrid, Spain; ^9^School of Computing, Edinburgh Napier University, Edinburgh, United Kingdom

**Keywords:** socio-cognitive computing, affective computing, social interaction, virtual and augmented reality, social robots, physiological computing

## 1. Introduction

Social Cognition focuses on how people process, store, and apply information about other people and social situations. It focuses on the role that cognitive processes play in our social interactions (Ostrom, 1984; Fiske and Taylor, 1991; Kunda, 1999). There has been increasing interest in the links between social cognition and brain function (Brothers, 1990). While substantial research has indicated that abilities such as theory of mind, social perception, attributional style, and emotion perception are clearly related to social outcomes (Pinkham et al., 2016), research has been slowed by problems in the measurement of these abilities.

Social interactions have been studied from physiological measurements for about 90 years (Riddle, 1925). The physiological basis of social constructs, processes and behavior has been widely investigated, firstly through social psychophysiology (Boyd and DiMascio, 1954; Kaplan and Bloom, 1960; Leiderman and Shapiro, 1964; Cacioppo and Petty, 1983), and later through social neuroscience (Brothers and Ring, 1992; Adolphs, 2001; Cacioppo and Berntson, 2002). Nowadays, Physiological Computing has emerged as a category of technology where electrophysiological data recorded directly from the human activity are used to interface with a computing device (Fairclough, 2009). This technology becomes even more relevant when computing can be integrated pervasively into everyday life environments.

“Physiological Computing of Social Cognition” should be understood as the application of physiological computing to the evaluation and/or treatment of social cognition abilities (Chanel and Mühl, 2015). It comprises a set of theoretical interdisciplinary frameworks, methodologies, methods, and hardware/software tools to interpret/act on how the human physiology mediates social interactions.

This Research Topic has provided a means for researchers and academicians who have a current or developing interest in the area of evaluation of social cognitive deficits and/or training in social cognition (e.g., cognitive enhancement therapies, attributional style training, social cognition and interaction training, or training of affect recognition, among others). Papers with a special focus on multidisciplinary approaches and multimodality were especially welcome. We finally accepted a total of six articles for this Research Topic.

## 2. The Papers

Of the six papers, three are directly related to the neural computation of brain activity. The first paper “Neural Correlates of Racial Ingroup Bias in Observing Computer-Animated Social Encounters” by Katsumi and Dolcos contributes on the neural correlates of intergroup processes and non-verbal perception by analyzing fMRI recordings. According to the authors, in spite of evidence for the role of group membership in the neural correlates of social cognition, the mechanisms associated with processing non-verbal behaviors shown by racially ingroup versus outgroup individuals remain unclear. Caucasian members experienced fMRI recording while watching social encounters with ingroup and outgroup characters showing dynamic and static non-verbal behaviors. The discoveries after computing the fMRI neuroimages shed light on the mechanisms of racial ingroup bias in observing social encounters and have suggestions for understanding elements identified with successful interactions with people from diverse backgrounds.

A second paper by Park et al. denominated “EEG Beta Oscillations in the Temporoparietal Area Related to the Accuracy in Estimating Others' Preference” explores brain activity through EEG signals identified with the forecast of the inclination of other persons through thin-slicing. This implies that humans often endeavor to judge what others prefer dependent on a narrow slice of experience. In each trial of the task, participants were demonstrated an image of either a target person or self, trailed by the introduction of a film poster over which participants anticipated the target person's preference as liking or disliking. The outcomes of EEG signal processing suggest that right temporoparietal beta oscillations might be correlated with one's capacity to predict what others prefer with minimal information.

Another neuroimaging-based article titled “Transcranial Direct Current Stimulation Altered Voluntary Cooperative Norms Compliance Under Equal Decision-Making Power” by Li et al. exhibits that anodal transcranial direct current stimulation (tDCS) enhances the voluntary cooperative norms compliance compared with the sham treatment, whereas cathodal tDCS breaks it down. The outcomes checked that voluntary cooperative norms compliance of all participants was altered by activating right dorsolateral prefrontal cortex (rDLPFC). The discoveries approve that enhancing the excitability of the rDLPFC utilizing tDCS prompts high consistence in voluntary cooperation and this impact is explicit to equal as opposed to unequal decision-making power.

In another article titled “The Two-Systems Account of Theory of Mind: Testing the Links to Social- Perceptual and Cognitive Abilities” by Meinhardt-Injac et al. theory of mind (ToM) is calculated through testing four domains of abilities: holistic face perception, face recognition, relational reasoning and language. This paper represents an achievement in the investigation of the respective roles of face recognition and language in the two-systems account of ToM. To test the particular roles of explicit abilities in both processes, the researchers administered 15 experimental procedures to a large number of participants, testing capacity in face recognition and holistic perception, language, and reasoning. ToM was estimated by a set of tasks expecting capacity to track and to induce others' complex emotional and mental states. The outcomes feature the respective roles of face recognition and language, in this manner contributing closer empirical specification of the two-systems framework.

The next paper “Eye Movements During Everyday Behavior Predict Personality Traits” by Hoppe et al. shows that a person's level of neuroticism, extraversion, agreeableness, conscientiousness, and perceptual curiosity can be anticipated only from eye movements recorded during an everyday task. The authors tracked eye movements and assessed personality traits using questionnaires. Utilizing artificial intelligence and a rich set of features encoding distinctive eye movement features, the researchers were able to reliably foresee four of the Big Five personality traits as well as perceptual interest.

The last paper “Emotion Regulation in the Prisoner's Dilemma: Effects of Reappraisal on Behavioral Measures and Cardiovascular Measures of Challenge and Threat” by Chu et al. associates cooperation and cardiovascular responses in people that were defeated by their rival in the first round of an iterated Prisoner's dilemma. The following cardiovascular were computed: heart rate, ventricular contractility, cardiac output, and total peripheral resistance. Participants were either primed with the emotion-regulation strategy of reappraisal or no-emotion-regulation, and their opponent either expressed an amused smile or a polite smile after the results were presented. The authors found that cooperation behavior diminished in the no-emotion-regulation group when the rival expressed an amused smile compared to a polite smile. In the cardiovascular measures, they found significant contrasts between the emotion regulation conditions utilizing the biopsychosocial model of challenge and threat.

## 3. Conclusion

The six papers together have earned more than 84,000 views as of August 10th, 2019. This confirms the popularity of physiological computing of social cognition as a subject for readers. We, the guest-editors of this Research Topic, trust the growing importance of physiological computing in areas as different as computer science, engineering, biology, psychology, medicine and neuroscience. Moreover, we believe that physiological computing has not exploited all its abilities and has not yet reached its full potential. In addition, the design of physiological computing applications in the field of social cognition is only at a starting moment (Fernández-Sotos et al., 2019; García et al., 2019). We foresee a vast growth of solutions using the social cognition/physiological computing binomial in the near future.

## Author Contributions

All the authors contributed equally to the writing of the editorial.

### Conflict of Interest Statement

The authors declare that the research was conducted in the absence of any commercial or financial relationships that could be construed as a potential conflict of interest.
